# Quantitative nuclear histomorphometric features are predictive of Oncotype DX risk categories in ductal carcinoma in situ: preliminary findings

**DOI:** 10.1186/s13058-019-1200-6

**Published:** 2019-10-17

**Authors:** Haojia Li, Jon Whitney, Kaustav Bera, Hannah Gilmore, Mangesh A. Thorat, Sunil Badve, Anant Madabhushi

**Affiliations:** 1 Department of Biomedical Engineering, Case Western Reserve University, Cleveland, OH USA; 2 University Hospitals Cleveland Medical Center, Cleveland, OH USA; 3 Centre for Cancer Prevention, Wolfson Institute of Preventive Medicine, Queen Mary University of London, London, UK; 4 School of Cancer & Pharmaceutical Sciences, Faculty of Life Sciences & Medicine, King’s College London, London, UK; 5 Department of Pathology and Laboratory Medicine, Indiana University, Indianapolis, IN USA

**Keywords:** Ductal carcinoma in situ, Oncotype DX, Quantitative histomorphometry, Nucleus

## Abstract

**Background:**

Oncotype DX (ODx) is a 12-gene assay assessing the recurrence risk (high, intermediate, and low) of ductal carcinoma in situ (pre-invasive breast cancer), which guides clinicians regarding prescription of radiotherapy. However, ODx is expensive, time-consuming, and tissue-destructive. In addition, the actual prognostic meaning for the intermediate ODx risk category remains unclear.

**Methods:**

In this work, we evaluated the ability of quantitative nuclear histomorphometric features extracted from hematoxylin and eosin-stained slide images of 62 ductal carcinoma in situ (DCIS) patients to distinguish between the corresponding ODx risk categories. The prognostic value of the identified image signature was further evaluated on an independent validation set of 30 DCIS patients in its ability to distinguish those DCIS patients who progressed to invasive carcinoma versus those who did not. Following nuclear segmentation and feature extraction, feature ranking strategies were employed to identify the most discriminating features between individual ODx risk categories. The selected features were then combined with machine learning classifiers to establish models to predict ODx risk categories. The model performance was evaluated using the average area under the receiver operating characteristic curve (AUC) using cross validation. In addition, an unsupervised clustering approach was also implemented to evaluate the ability of nuclear histomorphometric features to discriminate between the ODx risk categories.

**Results:**

Features relating to spatial distribution, orientation disorder, and texture of nuclei were identified as most discriminating between the high ODx and the intermediate, low ODx risk categories. Additionally, the AUC of the most discriminating set of features for the different classification tasks was as follows: (1) high vs low ODx (0.68), (2) high vs. intermediate ODx (0.67), (3) intermediate vs. low ODx (0.57), (4) high and intermediate vs. low ODx (0.63), (5) high vs. low and intermediate ODx (0.66). Additionally, the unsupervised clustering resulted in intermediate ODx risk category patients being co-clustered with low ODx patients compared to high ODx.

**Conclusion:**

Our results appear to suggest that nuclear histomorphometric features can distinguish high from low and intermediate ODx risk category patients. Additionally, our findings suggest that histomorphometric features for intermediate ODx were more similar to low ODx compared to high ODx risk category.

## Background

Ductal carcinoma in situ (DCIS) of the breast comprises a morphologically and biologically diverse group of cancerous lesions restricted to the breast ducts. The incidence of DCIS has seen a dramatic increase from 5.83 per 100,000 women in 1973 to 34.43 in 2014 [1]. One of the major causes for this increase appears to be the increasing prevalence of breast screening mammography [2], leading in turn to the discovery of these lesions at a much earlier time point. Approximately, 25% of all breast cancers in the USA are DCIS and 83% of all breast in situ cases diagnosed during 2010–2014 were DCIS, with the age- specific rate being highest in women between 65 and 75 years old (108.3 per 100,000 for 65–69 and 103.1 for 70–74) from 2010 to 2014 for carcinoma in situ [1].

With an estimated 1 out of every 33 women in the USA expected to suffer from DCIS during her lifetime [3], it becomes crucial to predict which of these women with DCIS might recur or progress to invasive breast cancer. Presently, the gold standard for treatment of DCIS is breast-conserving therapy, which includes a lumpectomy followed by adjuvant radiation therapy to remove the residual tumor. Hormonal therapy is also offered to patients with estrogen receptor (ER)-positive cancer. However, studies [4, 5] have found that radiotherapy (RT) can often be omitted in low-risk DCIS by demonstrating the RT did not have significant additional benefits to those patients. Since RT is relatively expensive, time-consuming, and often carrying significantly deleterious side effects [6], it is critical to identify DCIS patients with low recurrence risk to avoid the overtreatment.

Gene expression methods such as Oncotype DX (ODx) [7] DCIS recurrence score have been validated in being able to identify those DCIS patients in whom post-lumpectomy RT can be safely omitted. The ODx DCIS score leverages a panel of 12 genes including seven genes purely predictive of recurrence risk along with five reference genes. The output of the ODx DCIS assay is a score, scaled between 0 and 100. Three risk categories are then defined according to the scaled score: (1) low-risk (< 39), (2) intermediate-risk (39–54), (3) high-risk (55–100). Women with a low ODx score have a lower risk of recurrence than those with a high ODx risk score and may derive a lesser benefit from adjuvant RT. However, ODx for DCIS is limited by its high cost, limited availability, and being tissue-destructive. In addition, although a study [8] involving DCIS patients from multi-institutions has confirmed that ODx scores for risk stratification of DCIS patients provided valuable information to physicians and has effectively impacted treatment planning for DCIS patients, the actual prognostic meaning of the intermediate ODx risk category still remains unclear [7]. In clinical practice, DCIS patients with intermediate ODx risk scores tend to be considered high risk of recurrence for the purpose of treatment planning [9], which may potentially lead to an overtreatment.

Quantitative histomorphometry (QH) refers to the use of computerized methods and tools to quantitatively extract features of disease morphology from digitized images of tissue slides that may often be too subtle for visual discernment. QH enables an objective and reproducible measurement of the characteristics of the tumor at the sub-visual level, which is one of the ways to minimize the intra- and inter-observer variability that is often found in visual examination by pathologists [10]. QH features have shown to be independently prognostic across different cancer types including breast [11, 12], lung [13, 14], and oral cancer [15].

In this paper, we present a preliminary study to explore the potential role of quantitative nuclear histomorphometric features including nuclear shape, texture, and spatial arrangement from routine H&E-stained slide images of DCIS patients to distinguish between the high, intermediate, and low ODx DCIS risk categories. A total of *N* = 75 patients were retrospectively identified as having undergone surgical excision for DCIS and with a corresponding ODx DCIS score available. Using a combination of supervised classification and unsupervised clustering approaches, we sought to evaluate the ability of the features to discriminate between these DCIS patients with (1) high ODx vs. low ODx, (2) high ODx vs. intermediate ODx, (3) intermediate ODx vs. low ODx, (4) high and intermediate ODx vs. low ODx, and (5) high ODx vs. low and intermediate ODx risk categories. The prognostic value of the identified features was further evaluated on an independent validation set of 30 DCIS patients, in their abilities to distinguish the DCIS patients who progressed to invasive ductal carcinoma versus those who did not.

## Methods

**Fig. 1:**
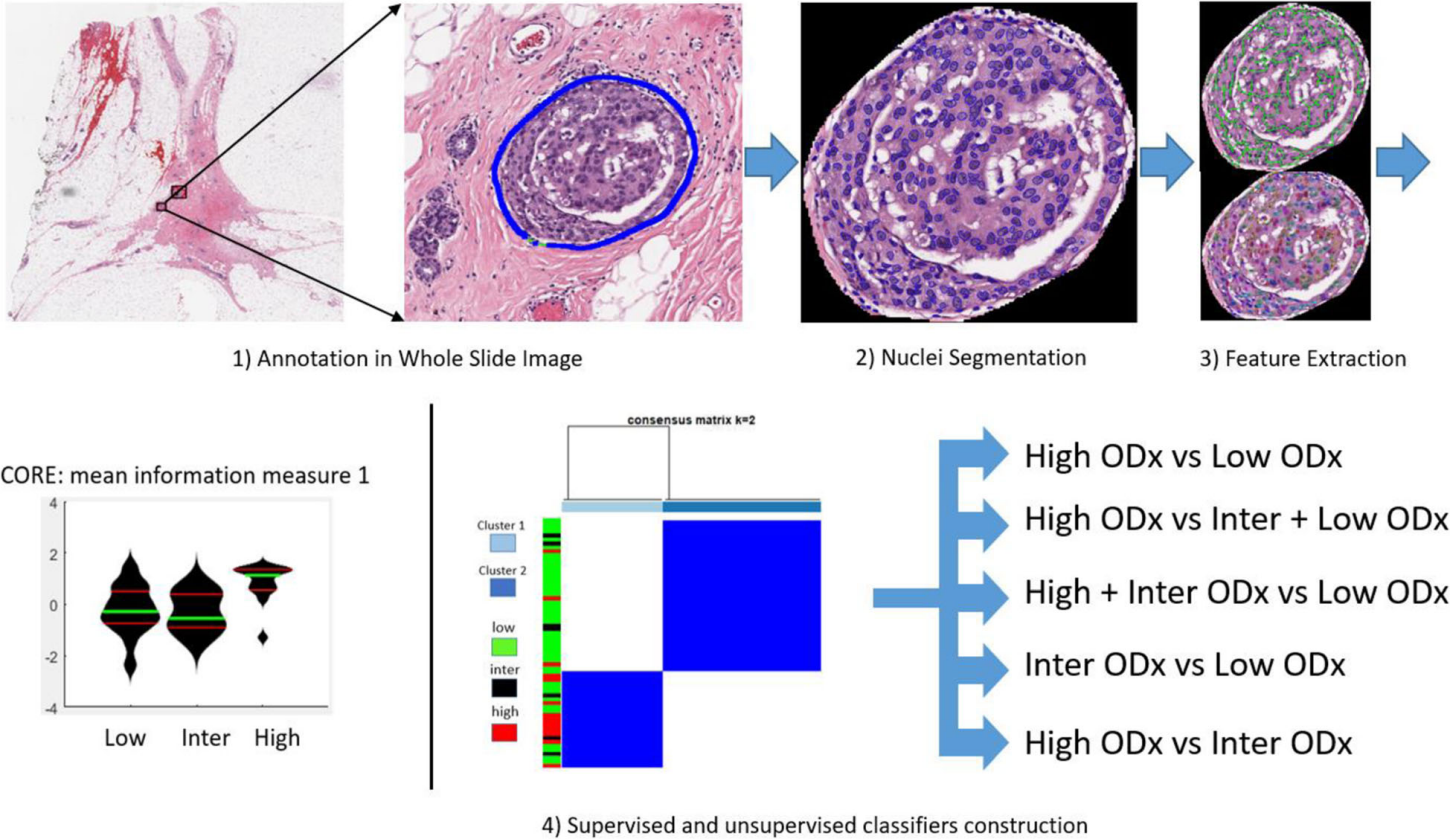
Illustration of the overall workflow: (1) Regions of DCIS were annotated on the whole slide image (WSI) by an experienced breast pathologist. (2) Nuclei were segmented from the annotated tumor region via a deep learning model [16]. (3) Nuclear histomorphometric features were extracted. (4) The features were then evaluated in their ability to distinguish different ODx risk categories via supervised classification and unsupervised clustering approaches

Figure 1 illustrates the overall workflow of the model construction and analysis.

### Data description

An IRB (institutional review board)-approved, retrospective chart review of women involved in a large DCIS retrospective study at the Indiana University from 2012 to 2016 yielded 75 patients who were diagnosed with pathologically confirmed DCIS and with a corresponding Oncotype DX DCIS score available in the final pathology report. Associated clinical information was also collected, following complete de-identification and anonymization of the patient studies. All protected health information (PHI) was scrubbed from the corresponding slides by an honest broker. The age group for these patients was 40–86. Out of the 75 patients, 8 were excluded because of non-salvageable biopsy tissue. We then digitized the H&E-stained biopsy of the surgical resected samples for the remaining 67 patients via whole slide scanners. Additionally, one slide image was subsequently excluded on account of digital slide scanning artifacts, one was excluded for having no stain, and three were excluded due to the limited tumor region found on the digitized tissue slide images (two corresponding to the low ODx risk category and one corresponding to the intermediate ODx risk category), leaving us with 62 analyzable tissue slide images (corresponding ODx scores included in Additional file 1: section S1) which formed the initial training/cross-validation set (D1 in Fig. 3). All the images were resized to × 20 to maintain a consistent magnification across all the slide images. The details on the tumor tissue slide preparation and scanning procedure are included in Additional file 2: section S2 under the heading “Digital Slide Acquisition Procedure”. Four tissue images for each of the three ODx risk categories are included in Additional file 3: section S3. The distribution plots for H&E staining intensity for each patient are included in S.2 as Fig. 1. In order to evaluate the prognostic ability of the QH features identified from D1, additionally, an external validation set of *n* = 30 (D2 in Fig. 3) from the UK/ANZ DCIS clinical trial, was retrieved and collected from Queen Mary University of London. The dataset comprised 15 DCIS patients who progressed to invasive ductal carcinoma and 15 DCIS patients without local recurrence or progression. The tissue slides in the validation set were scanned by Histech3D scanners at × 43 magnification, the images were then subsequently downsized to × 20 in order to keep the magnification consistent with the training set (D1).

**Fig. 2:**
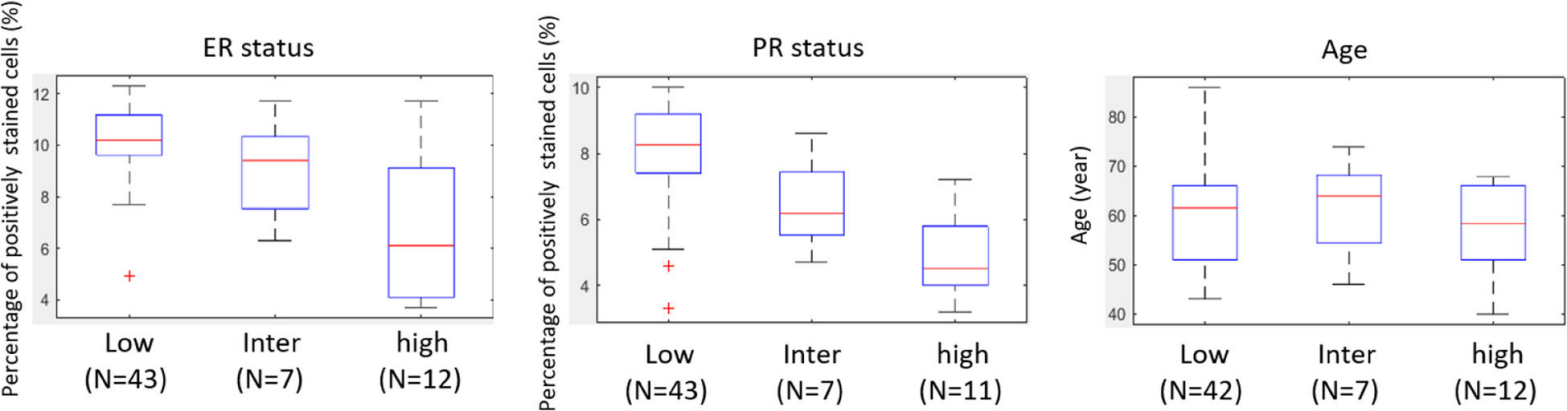
Distribution of clinical variables (ER status, PR status, and age) across the three ODx risk categories for patients in D1. Abbreviation: inter: intermediate

The available clinical variables on patients in D1 including age, ER, and progesterone receptor (PR) status (the percentage of positively stained cells) determined by immunohistochemistry staining were collected. According to the Wilcoxon rank sum test (WRST) [17], ER status for DCIS patients in low/intermediate ODx categories was significantly (*p* = 4.5e−05/*p* = 0.05) higher than the high ODx category, but no significant difference was observed between low versus intermediate ODx risk category. PR status however showed significant statistical differences between the different ODx risk categories (*p* = 0.03 for high vs. intermediate, *p* = 0.02 for low vs. intermediate, *p* = 1.3e−06 for high vs. low). This seems intuitive given that PR gene expression itself contributes to the DCIS ODx score calculation. The age spread for each risk group was comparable among the three ODx risk categories. The boxplots of ER status (low: 43, intermediate: 7, high: 12), PR status (low: 43, intermediate: 7, high: 11), and age (low: 42, intermediate: 7, high: 12) across the three ODx risk categories are shown in Fig. 2.

A multivariate analysis based on the three clinical variables with the ODx risk categories was also performed on D1 with the results included in S.2 as Table 1; no clinical variable was found to be significantly associated with the ODx risk categories in multivariate analysis.

**Table 1:** Summary of features examined. Feature family names (column 1), number of extracted features (column 2), and feature descriptions (column 3)

Feature family	Quantity	Feature description
Global Graph	51	Descriptors from Delaunay, Voronoi, and minimum spanning tree diagram
Shape	100	Nuclei area, smoothness, invariant moments and Fourier descriptors
Cell Orientation Entropy (CORE)	39	Disorder of neighbor nuclei polarity
Cell Cluster Graph (CCG)	25	Local subgraph connectivity
Haralick Texture	26	Relative pixel intensity, contrast entropy, and energy

**Fig. 3:**
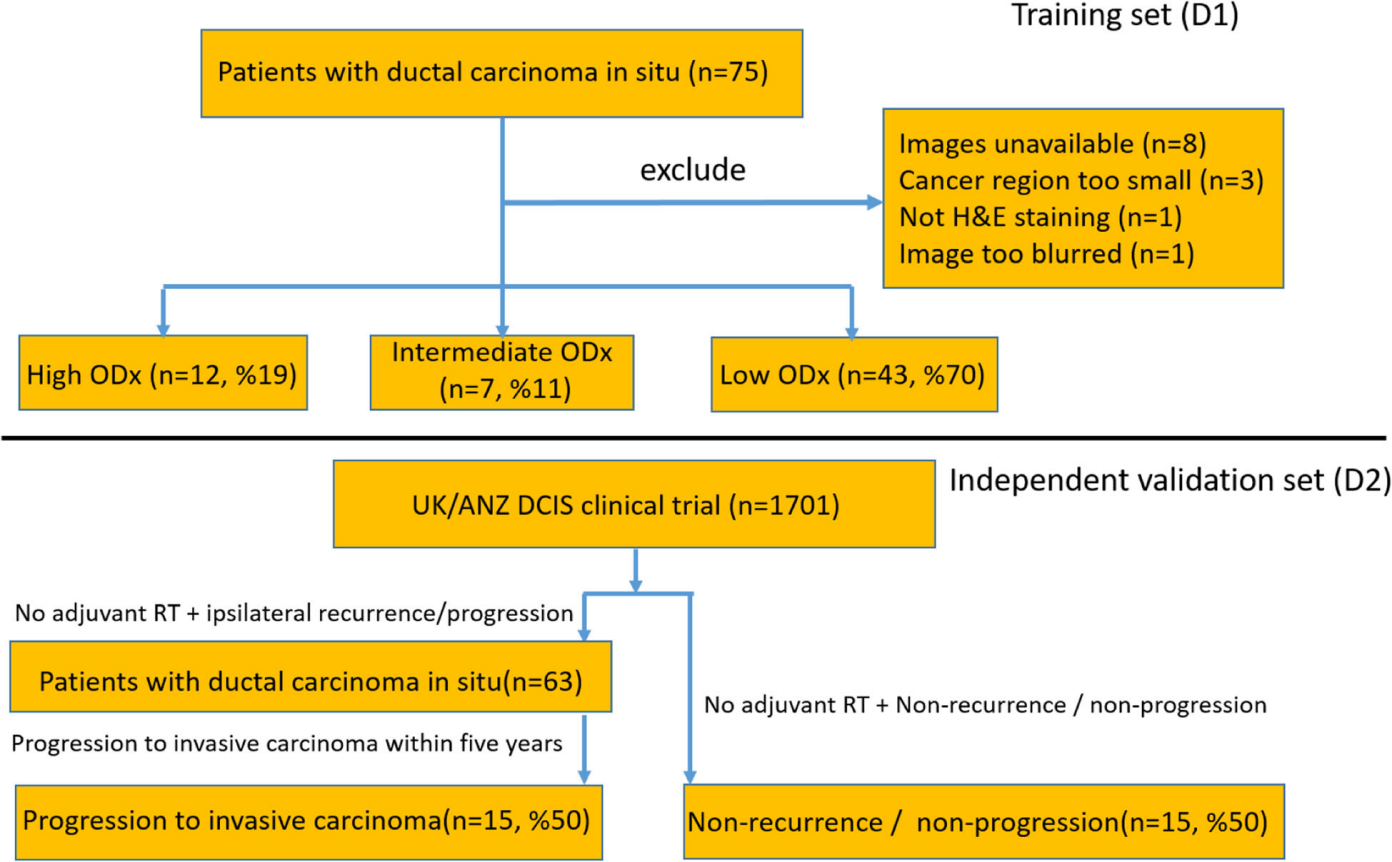
Inclusion and exclusion criteria for the patient slides selected for this study and the data distribution across the ODx risk categories for training set (D1). Patient distribution between the recurrence groups for the independent validation set (D2)

### Nuclei segmentation

The individual cancer cell nuclei within the noninvasive cancerous ducts were segmented from the whole slide image (WSI) at × 20 magnification using a deep learning model described in the work by Janowczyk et al. [16]. The deep learning model was trained on a dataset consisting of 141 breast cancer tissue images with individual nuclei manually annotated. The model with a nine-layer convolutional neural network structure was executed in Caffe framework using 32 × 32 sized patches on a Titan XGPU running CUDA 7.5. The deep learning model assigned each image pixel a likelihood of belonging to a nucleus, in turn resulting in a nuclei probability map. By selecting an appropriate threshold, the nuclei probability map images were converted into binary masks of the nuclei, using which the subsequent feature extraction was carried out. The quality of the nuclear segmentation approach was confirmed by selecting 10 images and visually inspecting the corresponding deep learning segmentation-derived contours against the corresponding manual contours.

### Feature extraction

**Fig. 4:**
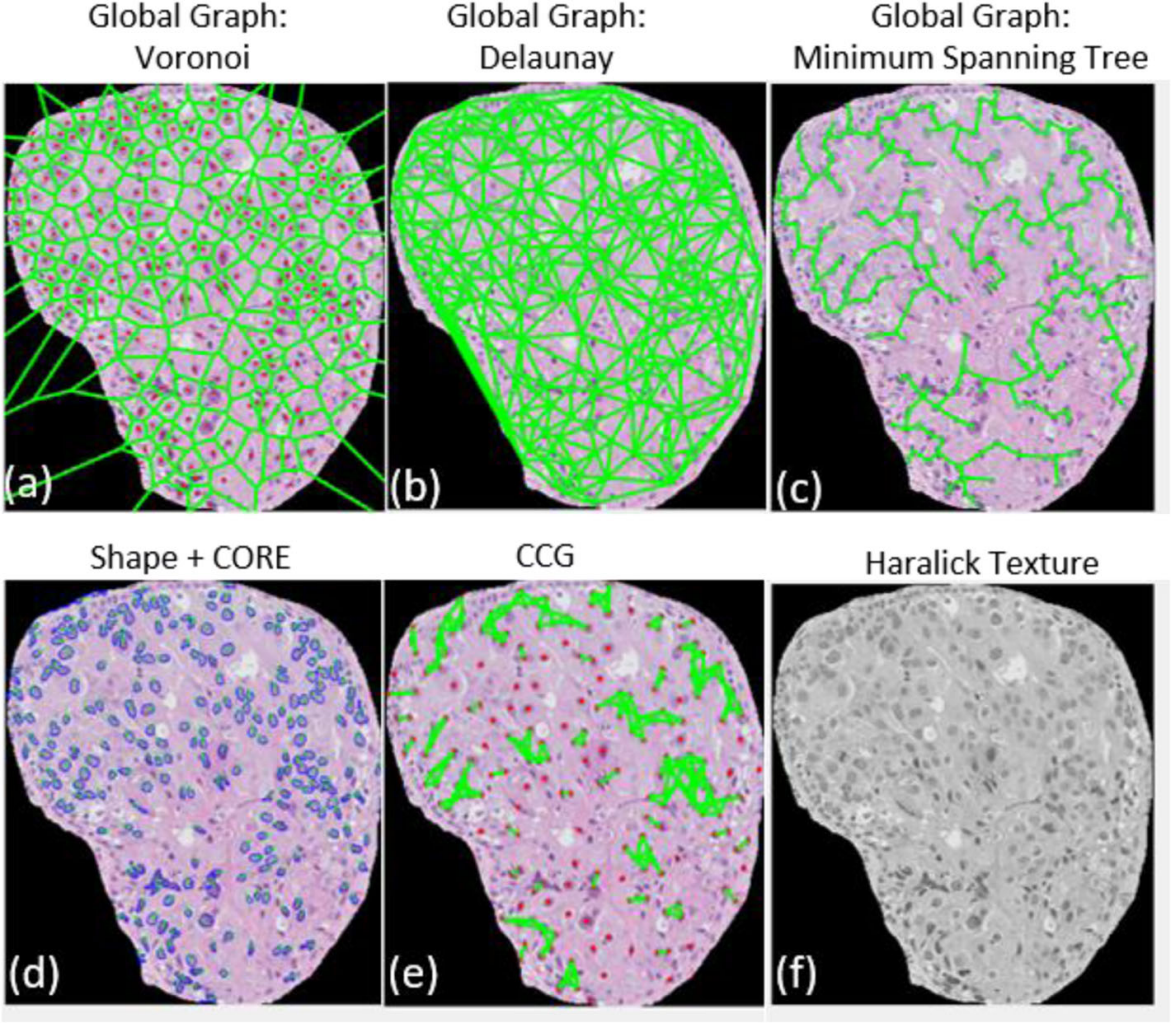
Illustration of the feature maps corresponding to global graph (Voronoi (**a**), Delaunay (**b**), and Minimum Spanning Tree (**c**)), Shape (**d**), CORE (**d**), CCG (**e**), and Haralick Texture (**f**) features, capturing respectively spatial arrangement, shape, orientation, local arrangement, and heterogeneity of nuclei within a tissue image of a DCIS patient corresponding to the high ODx risk category the feature maps corresponding to the intermediate- and low-risk categories were included in S.3 as Figure 2 (II) and Figure 2 (III) respectively

A total of 241 nuclei features (all the feature names listed in Additional file 4: section S4) were extracted from each of the WSIs. These corresponded to five feature families including Global Graph, Shape, Cell Cluster Graph (CCG) [18], Cell Orientation Entropy (CORE) [19], and Haralick Texture feature family (Fig. 4). A more detailed description of the features corresponding to each feature family are included in S.2 under the heading “Feature Description”.

### Model construction

#### Supervised classification

Four different supervised feature ranking methods were employed to identify the most discriminated features between the different ODx risk categories in D1. Those feature ranking methods were implemented in conjunction with three different supervised machine learning classifiers to build classification models capable of distinguishing the different ODx risk categories via a random sub-sampling-based cross validation (referred to as cross validation below).

The feature ranking methods include (1) covariance, (2) WRST [17], (3) minimum redundancy maximum relevance based on Mutual Information Difference (MRMR-mid) [20], and (4) minimum redundancy maximum relevance based on Mutual Information Quotient (MRMR-miq) [20]. The supervised machine learning classifiers (referred to as classifier below) include (1) support vector machine (SVM) [21], (2) linear discrimination analysis (LDA) [22], and (3) quadratic discriminant analysis (QDA) [23].

Specifically, in each cross validation, a training set was randomly subsampled from the whole dataset (D1), leaving the remaining subset as the testing set. Then each of the classifiers would be trained based on the top features identified from the training set by one of the four feature ranking methods and was validated on the testing set. Additionally, in each round, a Bhattacharyya (BC) distance between the top identified feature set in the testing set corresponding to the two classes was calculated by averaging the BC distances for the individual top features. The cross validation was repeated for 100 rounds for each combination of feature ranking method and classifier with the area under the receiver operating characteristic curve (AUC) calculated to evaluate the model performance in each round. The overall model performance was represented by the averaged AUC value over 100 repetitions of cross validation. The BC distance array comprising 100 distance values from each run of cross validation were also averaged across the 100 repetitions.

#### Unsupervised clustering

Principal component analysis (PCA) [24] was employed to identify the principal components from the PCA feature space and worked in conjunction of an unsupervised clustering approach to distinguish between DCIS patients corresponding to different ODx risk categories.

### Statistical analysis

For the supervised classification strategy, we employed four different feature selection methods. (1) Covariance was used to identify the most correlated features with the ODx risk category based on the corresponding correlation coefficient value. (2) WRST ranks features based on their corresponding *p* value associated with the null hypothesis, the null hypothesis being that the feature distribution in one class has an equal median value as in the other class [17]. (3) MRMR-mid and (4) MRMR-miq approaches identify features that not only are highly discriminatory between the two classes of interest, but also are minimally correlated with each other [20]. In addition, we used BC distance [25] to measure separation of the top identified feature set between two defined classes. The BC distance reflects the relative similarity between two statistical samples, which is in turn derived from the Bhattacharyya coefficient assessing the overlap between the two probability distributions.

For unsupervised clustering, PCA was employed for feature selection. PCA converted original features into uncorrelated variables via an orthogonal transformation [24]. The top principal components, which in turn represent the majority of the feature variance in the original feature space, could be identified from the transformed PCA space.

For supervised classification, three different classifiers were utilized including LDA, QDA, and SVM. LDA and QDA model the distribution of the top QH features for each of the two classes, and then Bayes’ optimal solution was used to predict the class label for each patient given the corresponding top feature set [22, 23]. LDA additionally assumes equal covariance among the top identified features between the two output classes [22]. SVM is a discriminative classifier which outputs an optimal hyperplane representing the largest separation of the top identified features between the two classes [21].

Apart from the supervised classifiers, we also employed an unsupervised consensus clustering approach [26], which employed hierarchical clustering on the top two principal components from the PCA transformed feature space. The distance metric employed in hierarchical clustering was measured by the Pearson correlation coefficient. The clustering is performed in conjunction with a patient resampling rate of 0.8 over 50 runs to validate the stability of the principal components and their distinguishability between the defined classes for different classification tasks. After the 50 repeated clusterings, each patient is assigned a cluster group index (cluster 1 or cluster 2).

### Experimental design

#### Experiment 1: Evaluating the ability of nuclear histomorphometric features in distinguishing different ODx risk categories for DCIS

This experiment comprised the following classification tasks performed on D1 dataset, evaluating the ability of nuclear histomorphometric features in distinguishing (1) high ODx vs. low ODx, (2) high ODx vs. intermediate ODx, and (3) intermediate ODx vs. low ODx risk categories.

For each of the classification tasks, three top features were identified via each of the four different feature ranking methods (covariance, WRST, MRMR-mid, MRMR-miq) on the subsampled training set in the cross validation. Only three top features were used in order to prevent overfitting of the classifier on the training set [27]. The selected top feature set was then employed to train each of the three classifiers (LDA, QDA, SVM), and each of the trained classifiers was subsequently evaluated via AUC on the remaining testing set during each run of cross validation. The cross validation was repeated across 100 iterations to yield an average AUC for each of the 12 combinations of feature ranking methods and classifiers*.* In order to avoid the model biasing towards the majority class due to imbalanced class distributions [28] across the three ODx risk categories in D1, we subsampled the training set with an equal number of instances from each of the two defined classes. This class balance was maintained during all rounds of cross validation. Additionally, we utilized AUC to evaluate the model performance via cross validation, an approach that is less susceptible to testing set imbalance [29]. The training and testing set split during cross validation for each of the three classification tasks is listed in Table 2 (columns 2–4).

**Table 2:** Training and testing split in the cross validation for each of the five different classification tasks performed on the patients in D1

Classification tasks	High vs. low		High vs. inter		Inter vs. low		High + inter vs. low		High vs. low + inter	
ODx risk category	High	Low	High	Inter	Inter	Low	High/inter	Low	High	Low/inter
# of train/# of test	7/5	7/36	4/8	4/3	4/3	4/39	10/9	10/33	7/5	7/43
Total # of patients	55		19		50		62		62	

*Abbreviation*: *inter* intermediate, # number

Additionally, for each of the three classification tasks, the averaged BC distance [25] of the identified top feature sets between the two ODx risk categories was calculated across the repeated runs of cross validation, the approach was described in the “Model construction” section. Following the distance calculation, we utilized WRST to assess whether there was a statistically significant difference between the BC distance arrays for the three classification tasks.

Besides the cross validations with supervised feature ranking methods and classifiers, for each of the three classification tasks, we also performed PCA on the whole feature space of all involved patients to identify the first two principal components, which in turn represent the maximum information present in the original feature space [24]. An unsupervised clustering method we have described in “Statistical analysis” section was then implemented with the identified principal components on the patients for each of classification tasks to evaluate the clustering of ODx risk categories.

#### Experiment 2: Evaluating difference between nuclear histomorphometric features for intermediate versus low and high ODx risk categories

In this experiment, we sought to evaluate the similarity between nuclear histomorphometric features for two supervised classification tasks, which were also performed on D1 dataset, namely (1) high + intermediate ODx vs. low ODx and (2) high ODx vs. intermediate ODx + low ODx risk categories. We also employed unsupervised clustering to evaluate the relative proximity between the patients corresponding to high, intermediate, and low ODx risk categories.

Experiment 2 followed a similar design to Experiment 1 in terms of feature selection, supervised-, and unsupervised-based evaluation of the identified top features. In the supervised setting, the training and testing set split in cross validation for each of the two classification tasks is listed in Table 2 (columns 5–6). Additionally, in Experiment 2, the extracted nuclear histomorphometric features from all the patients in D1 were transformed into their corresponding PCA space. Subsequently, the unsupervised clustering was performed with the first two components in the PCA-transformed space on the whole D1 dataset.

#### Experiment 3: Validate the prognostic ability of the identified nuclear histomorphometric features on an independent validation set

In this experiment, we evaluated the prognostic value of the image features that were identified as being associated with ODx risk categories for the high vs. low ODx, high vs. intermediate ODx risk categories in Experiment 1, and for the high vs. intermediate + low ODx risk categories in Experiment 2 on D2 (*n* = 30; 15 DCIS patients who progressed to invasive cancer and 15 DCIS patients without any recurrence/progression). Unsupervised clustering was also performed on the set of image features to evaluate its ability to differentiate between DCIS patients who are and are not at risk for recurrence.

## Results

### Experiment 1: Evaluating the ability of nuclear histomorphometric features in distinguishing different ODx risk categories for DCIS

#### Experiment 1A: High ODx vs. low ODx

**Fig. 5:**
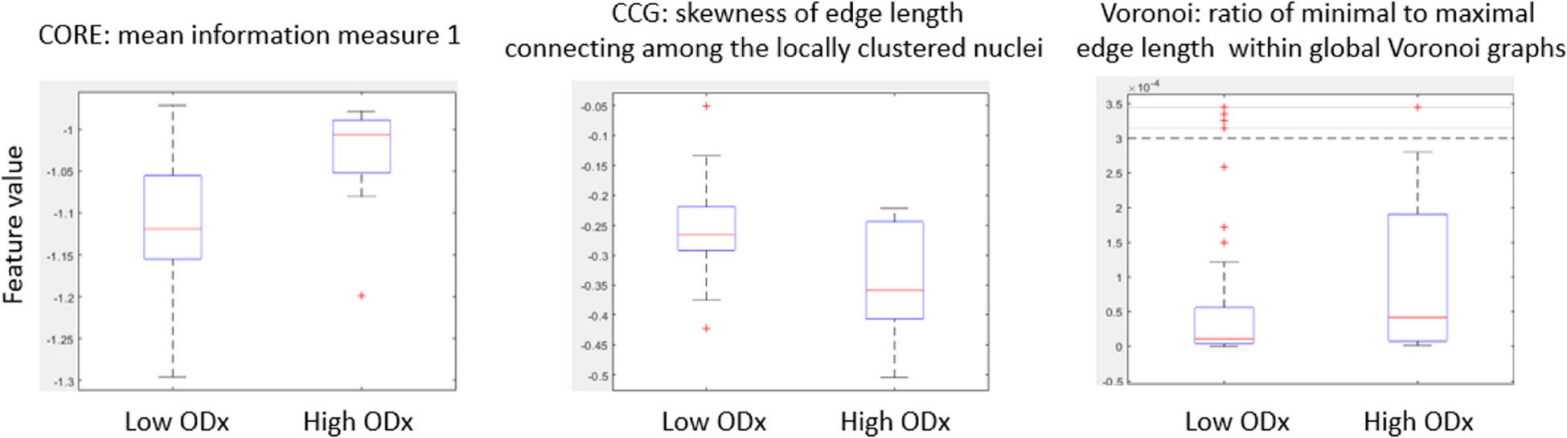
Value distribution of features most frequently appearing in top feature sets from classification task of high versus low ODx. The red lines in the plots represent the median of each population, and the upper and lower box bounds correspond to the 25th and 75th percentiles of the feature value distribution

The highest average AUC across different combinations of feature ranking methods and classifiers was 0.68 for the combination of MRMR-mid and SVM (Table 3 (italics in column 3)). The top three identified features, which most frequently appeared in the top feature sets over the 100 repetitions of cross validation, corresponded to disorder in the polarity of the individual nuclei (CORE: mean information measure 1), variation in spatial arrangement of locally clustered nuclei (CCG: skewness of edge length connecting among the locally clustered nuclei), range in the global arrangement of nuclei across the pathology slide image (ratio of minimal to maximal edge length within global Voronoi graphs). The corresponding boxplots for these features are illustrated in Fig. 5. The average values of BC distance between the top discriminating feature set corresponding to the high and that corresponding to low ODx risk categories are listed in Table 4 (column 2). Figure 8a illustrates the results of the unsupervised clustering with the first two PCA components from the PCA transformed feature space for the low and high ODx risk category patients. Three out of 12 patients corresponding to the high ODx risk category were embedded within cluster 2, and 11 out of 43 patients corresponding to the low ODx risk categories were embedded within cluster 1.

**Table 3:** Average AUCs (+ standard deviation) across 100 repetitions of cross validation from the models corresponding to different combinations of three classifiers (column 1) and four feature ranking methods (column 2) for five different classification tasks (columns 3–6: Experiment 1A, 1B, 1C, 2A, and 2B)

Classifiers	Feature ranking methods	High vs. low	High vs. intermediate	Intermediate vs. low	High + intermediate vs. low	High vs. low + intermediate
SVM	Covariance	0.66 ± 0.11	0.64 ± 0.19	0.56 ± 0.15	0.62 ± 0.08	0.65 ± 0.12
	WRST	0.64 ± 0.11	0.61 ± 0.16	0.55 ± 0.14	0.61 ± 0.08	*0.66 ± 0.12*
	MRMR-mid	*0.68 ± 0.12*	*0.67 ± 0.18*	0.53 ± 0.17	0.61 ± 0.10	0.63 ± 0.13
	MRMR-miq	0.67 ± 0.13	0.65 ± 0.17	0.54 ± 0.17	0.62 ± 0.10	0.64 ± 0.12
LDA	Covariance	0.65 ± 0.11	0.62 ± 0.19	*0.57 ± 0.15*	*0.63 ± 0.09*	0.65 ± 0.09
	WRST	0.63 ± 0.12	0.61 ± 0.18	0.53 ± 0.16	0.62 ± 0.08	0.64 ± 0.12
	MRMR-mid	0.65 ± 0.13	0.66 ± 0.19	0.54 ± 0.15	0.61 ± 0.09	0.64 ± 0.12
	MRMR-miq	0.64 ± 0.13	0.64 ± 0.21	0.55 ± 0.15	0.61 ± 0.09	0.66 ± 0.12
QDA	Covariance	0.62 ± 0.13	0.60 ± 0.18	0.54 ± 0.15	0.61 ± 0.09	0.64 ± 0.12
	WRST	0.59 ± 0.13	0.59 ± 0.15	0.52 ± 0.16	0.62 ± 0.09	0.63 ± 0.11
	MRMR-mid	0.61 ± 0.14	0.61 ± 0.17	0.51 ± 0.15	0.58 ± 0.11	0.62 ± 0.14
	MRMR-miq	0.63 ± 0.12	0.62 ± 0.18	0.53 ± 0.16	0.60 ± 0.10	0.63 ± 0.12

**Table 4:** Average BC distance between the top feature sets identified by four different feature ranking methods for each of the five classification tasks (Experiment 1A, 1B, 1C, 2A, and 2B) across 100 iterations of cross validation

Feature ranking methods	High vs. low	High vs. intermediate	Intermediate vs. low	High + intermediate vs. low	High vs. low + intermediate
Covariance	0.50	0.62	0.45	0.38	0.47
WRST	0.48	0.55	0.41	0.38	0.47
MRMR-mid	0.47	0.54	0.40	0.36	0.45
MRMR-miq	0.49	0.63	0.42	0.37	0.48

#### Experiment 1B: High ODx vs. intermediate ODx

**Fig. 6:**
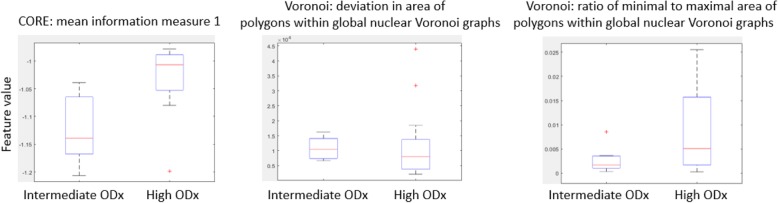
Value distribution of features most frequently appearing in top feature sets from classification task of high versus intermediate ODx. The red lines in the plots represent the median of each population, and the upper and lower box bounds correspond to the 25th and 75th percentiles of the feature value distribution

The highest average AUC across different combinations of feature ranking methods and classifiers was 0.67 for combination of MRMR-mid and SVM (Table 3 (italics in column 4)). The top three identified features corresponded to disorder in the polarity of the individual nuclei (CORE: mean information measure 1), variation in the global arrangement of nuclei (deviation in area of polygons within global nuclear Voronoi graphs), and range in the global arrangement of nuclei across the pathology slide image (ratio of minimal to maximal area of polygons within global nuclear Voronoi graphs). The corresponding boxplots for these features are illustrated in Fig. 6. Again, the average values of BC distance between the top feature set corresponding to the high ODx and that corresponding to intermediate ODx risk categories are listed in Table 4 (column 3). The results of the unsupervised clustering with the first two PCA components are illustrated in Fig. 8b with 5 out of 7 patients corresponding to the intermediate ODx risk category embedded within cluster 2 and 9 out of 12 patients corresponding to the high ODx risk category embedded within cluster 1.

#### Experiment 1C: Intermediate ODx vs. low ODx

The highest average AUC across different combinations of feature ranking methods and classifiers was 0.57, for the combination of Covariance and LDA (Table 3 (italics in column 5)). The average values of BC distance between the top discriminating feature set corresponding to the intermediate and that corresponding to the low ODx risk categories are listed in Table 4 (column 4). Figure 8c illustrates the unsupervised clustering results with the first two principal components from the PCA transformed feature space for the low and intermediate ODx risk category patients. The patients corresponding to the intermediate ODx risk category are nearly evenly embedded in the two clusters with 3 in cluster 1 and 4 in cluster 2.

WRST was implemented to evaluate the statistical difference between the BC distance arrays obtained from the three supervised classification tasks (Experiments 1A, 1B, and 1C) with *p* values listed in Table 5 (columns 2–3).

**Table 5:** *p* values from WRST implemented to compare the BC distance arrays obtained from classification task of Experiments 1A with 1C (column 2), classification task of Experiments 1B with 1C (column 3), and the classification task of Experiments 2A with 2B (column 4)

Feature ranking methods	High vs. low versus Low vs. intermediate	High vs. inter versus Lowvs. intermediate	High + intermediate vs. low versus High vs. intermediate + low
Covariance	0.026	0.0003	0.001
WRST	0.011	0.0005	0.002
MRMR-mid	0.017	1.9e−05	0.0005
MRMR-miq	0.022	5.6e−8	1.2e−05

### Experiment 2: Evaluating difference between nuclear histomorphometric features for intermediate versus low and high ODx risk categories

#### Experiment 2A: High + intermediate ODx vs. low ODx

The highest average AUC across different combinations of feature ranking methods and classifiers was 0.63, for the combination of covariance and LDA (Table 3 (italics in column 6)). The average values of BC distance between the top discriminating feature set corresponding to the high plus intermediate ODx and that corresponding to low ODx risk categories are listed in Table 4 (column 5).

#### Experiment 2B: High ODx vs. intermediate + low ODx

**Fig. 7:**
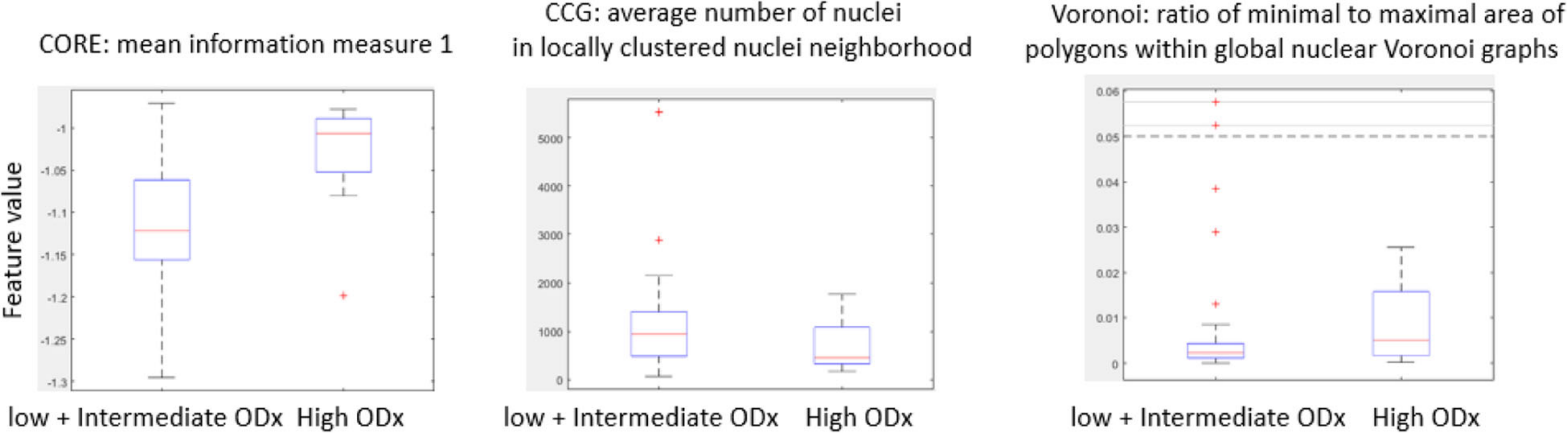
Value distribution of features most frequently appearing in top feature sets from classification task of high versus intermediate + low ODx. The red lines in the plots represent the median of each population, and the upper and lower box bounds correspond to the 25th and 75th percentiles of the feature value distribution

The highest average AUC across different combinations of feature ranking methods and classifiers was 0.66, for the combination of WRST and SVM and the combination of MRMR-miq and LDA (Table 3 (italics in column 7)). The top three identified features corresponded to disorder in the polarity of the individual nuclei (CORE: mean information measure 1), connectivity in local nuclei neighborhood (CCG: average number of nuclei in locally clustered nuclei neighborhood), range in the global arrangement of nuclei across the pathology slide image (ratio of minimal to maximal area of polygons within global nuclear Voronoi graphs). The corresponding boxplots for these features are illustrated in Fig. 7. The average values of the BC distance between the top discriminating feature set corresponding to the high and that corresponding to intermediate plus low ODx risk categories are listed in Table 4 (column 6).

WRST was implemented to evaluate the difference between the BC distance arrays obtained from the two supervised classification tasks (experiment 2A, experiment 2B) with *p* values listed in Table 5 (column 4).

#### Experiment 2C: High vs. intermediate vs. low ODx

**Fig. 8:**
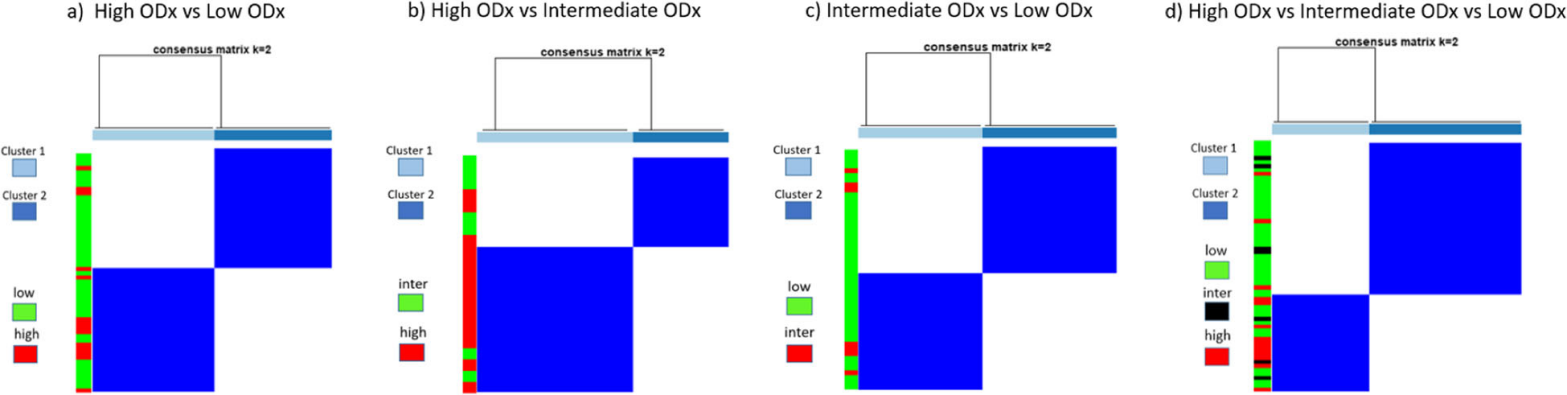
Unsupervised clustering (*k* = 2) utilizing the top 2 principal components from PCA-transformed feature space. From left to right are the plots corresponding to **a** high versus low ODx, **b** high versus intermediate ODx, **c** intermediate versus low ODx, and **d** high versus intermediate versus low ODx

**Fig. 9:**
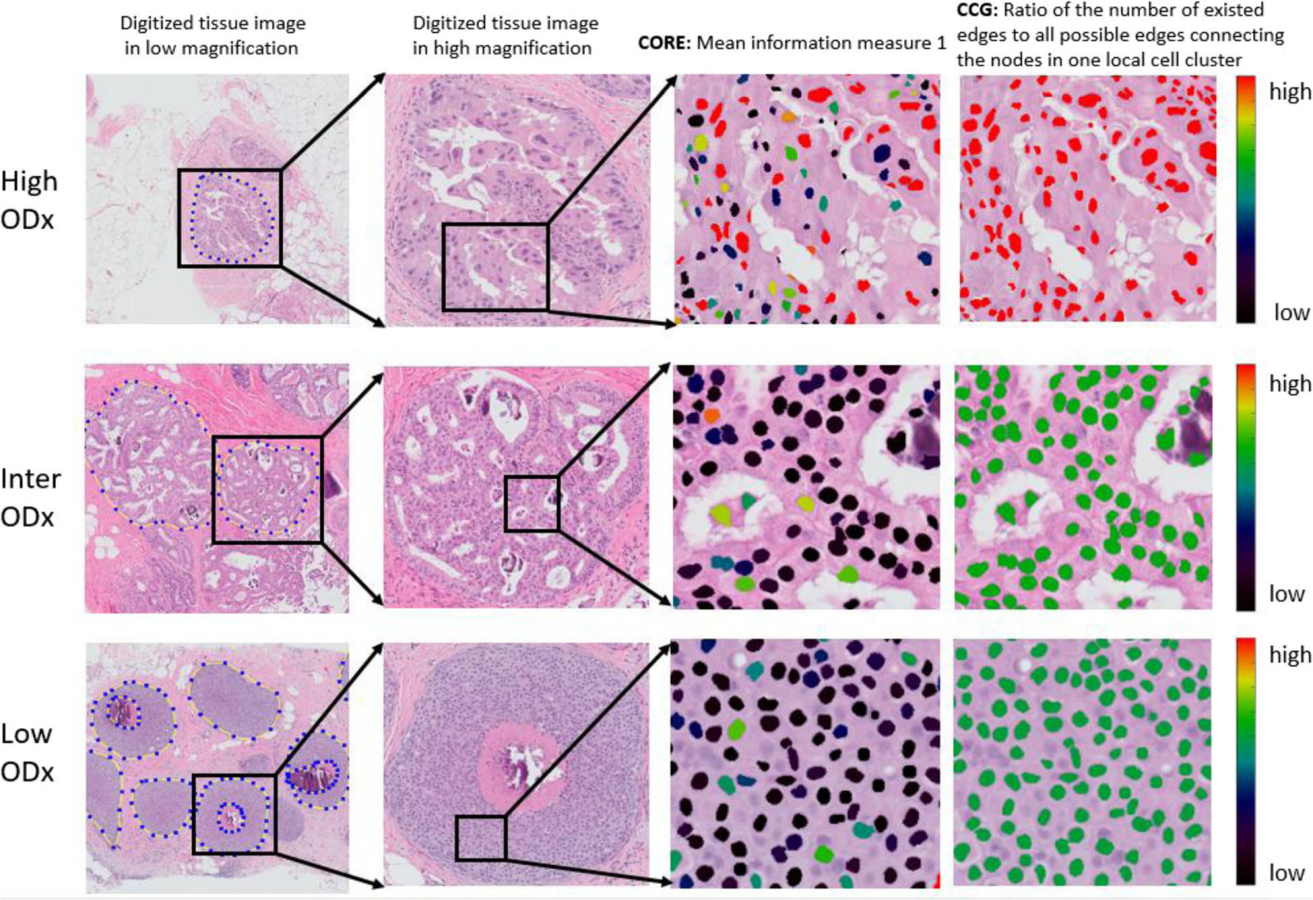
Representation of the top features. Left: Low-magnification view of breast ducts. Center left: boxed region brought into focus for greater magnification. Center right: × 20 magnification with nuclei highlighted with their corresponding top CORE feature value indicated by nuclei color. Right: × 20 magnification with nuclei highlighted with their corresponding top CCG feature value indicated by nuclei color

Results of the unsupervised clustering with the first two principal components from the PCA-transformed feature space for the low, intermediate, and high ODx risk category patients are illustrated in Fig. 8d. Thirty-two out of 43 patients corresponding to the low ODx risk category were embedded within cluster 2, and 9 out of 12 patients corresponding to the high ODx risk category were embedded within cluster 1. For the patients corresponding to the intermediate ODx risk category, 4 out of 7 patients were embedded within cluster 2, together with a majority of patients corresponding to the low ODx risk category (Fig. 9).

WRST was implemented to select the most distinguishable features between high and low ODx risk categories from each of the five feature families. The names of identified top features and *p* values from WRST implemented for different comparisons (high vs. low ODx, high vs. intermediate OD, low vs. intermediate ODx) are listed in Table 6. The features (in italics) identified to distinguish between high and low ODx risk categories were also found to discriminate between patients corresponding to the high and intermediate ODx risk category (*p* < 0.1). However, these features were unable to discriminate between patients corresponding to the low and intermediate ODx risk categories.

**Table 6:** List of most discriminating features between high and low ODx risk category for each feature family and corresponding *p* values from WRST for each of the different comparisons

Feature family	Feature name	High vs. low ODx	High vs. intermediate ODx	Low vs. intermediate ODx
*Global Graph*	*Average number of neighbor nuclei in a distance of a 50-pixel radius corresponding to each cancer nucleus*	0.007	0.08	0.3
Shape	Symmetry of nuclei shape	0.005	0.3	0.3
*CCG*	*The ratio of the number of existed edges to all possible edges connecting the nodes in one local cell cluster*	0.001	0.08	0.6
*CORE*	*Mean information measure1*	0.0002	0.005	0.7
*Texture*	*Standard deviation of pixel-wise gray-level distribution across nuclei*	0.001	0.05	0.5

From Table 3, we are able to observe that, independent of the combination of feature ranking method and classifier, significantly better discrimination between high and low/intermediate ODx risk categories was observed as compared to between low and intermediate ODx risk categories (based on the AUC value). This trend was observed across different combinations of feature ranking methods and classifiers.

The results in Table 4 and Table 5 suggested that the BC distance between high and low ODx risk category and between high and intermediate ODx risk category were significantly higher as compared to the corresponding distance between the intermediate and low ODx risk categories across all four feature ranking methods. Additionally, the BC distance between high and low + intermediate ODx risk categories was significantly higher compared to the separation observed between high + intermediate ODx and low ODx risk categories. The visual representation for two of the distinguishable features between high and low ODx risk categories was illustrated in Fig. 9.

#### Experiment 3: Validate the prognostic ability of the identified nuclear histomorphometric features on an independent validation set (D2)

**Fig. 10:**
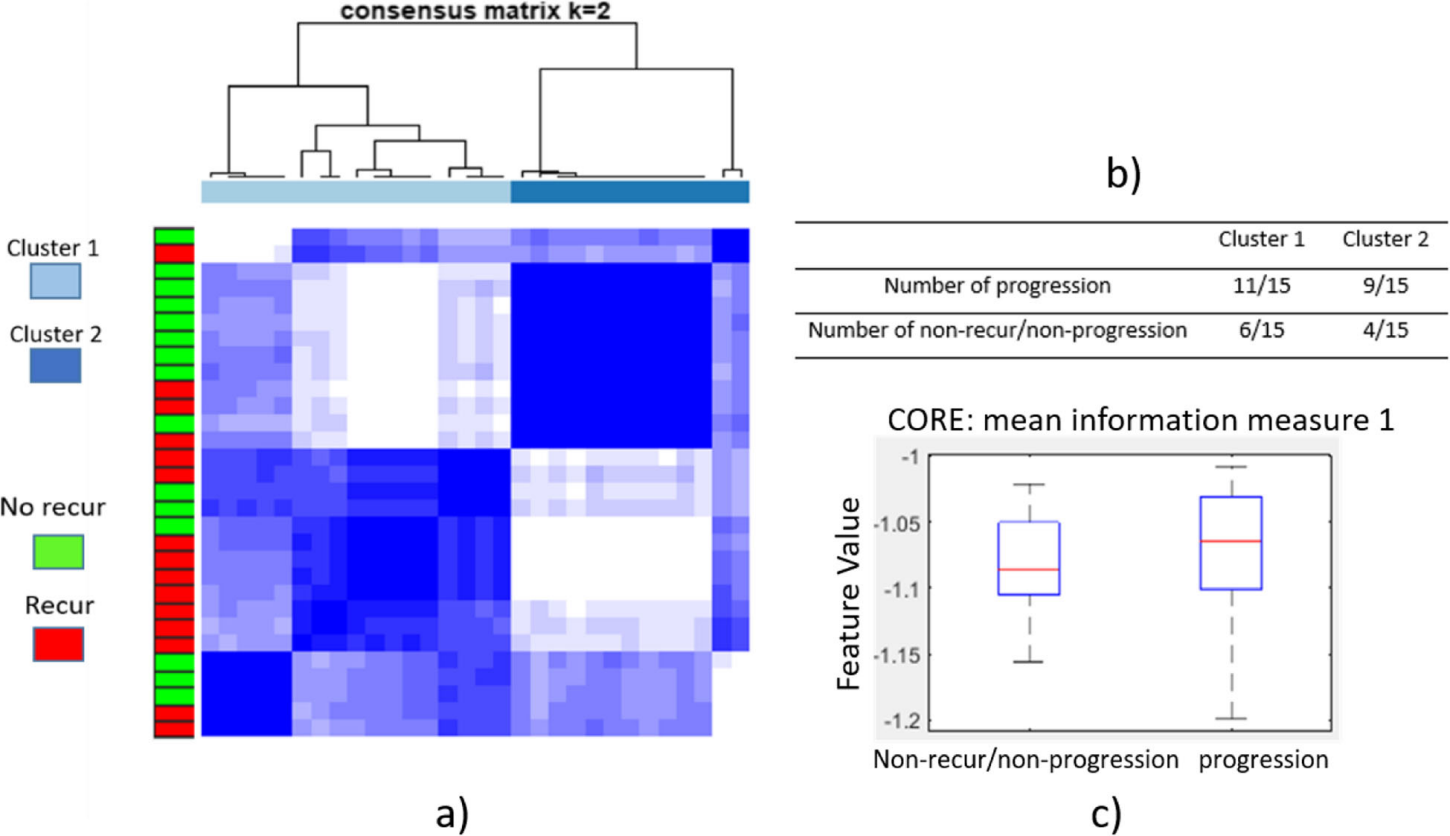
**a** The unsupervised clustering (*k* = 2) utilizing the histomorphometric signature learnt based on Experiments 1 and 2 (distinguishing low from high ODx risk categories, distinguishing intermediate from high ODx risk categories, and distinguishing low plus intermediate from high ODx risk categories). **b** Distribution of outcome of patients in cluster 1 and cluster 2 identified from unsupervised clustering. **c** Feature distribution (CORE: mean information measure 1) for the patients without recurrence/progression and patients with progression to invasive breast cancer. The red lines in the plots represent the median of each population, and the upper and lower box bounds correspond to the 25th and 75th percentiles of the feature value distribution

The cluster plot derived using the histomorphometric signature learnt based on Experiments 1 and 2 (distinguishing low from high ODx risk categories, distinguishing intermediate from high ODx risk categories and distinguishing low + intermediate from high ODx risk categories) is presented in Fig. 10a and the corresponding patient distribution across the progression to invasive cancer and non-recurrence/non-progression categories for each cluster identified from unsupervised clustering is shown in Fig. 10b. Eleven out of 15 DCIS patients who progressed to invasive ductal carcinoma and 6 out of 15 patients with non-recurrent/non-progressive cancer were grouped into one cluster (65% progressed to invasive cancer), while 4 out of 15 cases with progression to invasive cancer and 9 out of 15 non-recurrent/non-progressive cases were distributed in the other cluster (69% non-recurrent/non-progressive). Also as shown in Fig. 10c, feature values of average information measure 1 are higher in DCIS patients with progression compared to patients without recurrence/progression. This trend is consistent with the feature values observed in the high ODx risk category as compared to the low and intermediate ODx risk categories.

Specifically, the image signature consists of mean information measure 1 in CORE feature family; skewness of edge length connecting among the locally clustered nuclei in CCG feature family; ratio of minimal to maximal edge length within global Voronoi graphs in Global Graph feature family; deviation in area of polygons within global nuclear Voronoi graphs in Global Graph feature family; ratio of minimal to maximal area of polygons within global nuclear Voronoi graphs in Global Graph feature family; and average number of nuclei in locally clustered nuclei neighborhood in CCG feature family.

## Discussion

Multiple clinical trials including E5194 [4] and RTOG9804 [5] have shown that low-risk DCIS patients tend to receive minimal benefit from adjuvant RT. There is a clear unmet need to identify those DCIS patients with a relatively low likelihood of recurrence or progression to avoid the side effect [6] of unnecessary adjuvant RT for those patients. Oncotype DX (ODx) for DCIS is a gene expression-based assay to assess the recurrence risk of DCIS. While the ODx-derived risk category has been validated against the outcome on a cohort comprising 670 DCIS patients from ECOG E5194, it was not perfectly correlated [7]. In addition, the ODx test for DCIS is expensive, tissue-destructive, and requires specialized facilities. Another issue with the ODx DCIS test is the lack of true prognostic meaning and significance associated with patients assigned to the intermediate-risk category [7]. A lot of these intermediate ODx risk category patients may end up receiving adjuvant therapy and hence potentially be over-treated [9].

In this paper, we identified a set of image features associated with the different ODx risk categories. Additionally, the prognostic ability of these image features to predict DCIS with progression to invasive cancer versus DCIS without recurrence/progression was evaluated on a small independent test set (*n* = 30) of women with DCIS. Additionally, we sought to elucidate the morphologic attributes of the Oncotype DCIS intermediate category, a risk category with somewhat ambivalent prognostic significance (unlike the low and high ODx risk categories).

According to the results based on the supervised classifiers and unsupervised clustering for distinguishing high ODx vs. low ODx and high ODx vs. intermediate ODx, high ODx risk category was found to be distinguishable from low and intermediate ODx risk categories in terms of nuclear histomorphometric features. The top features identified as being discriminating of high ODx from intermediate plus low ODx risk categories included (1) mean information measure 1 of correlation between neighbor nuclei orientations, which captures the information pertaining to the disorder in the polarity of the individual nuclei; (2) the ratio of the number of existed edges to all possible edges connecting the nodes in one local cell cluster, reflecting the spatial arrangement of nuclei in locally clustered nuclei neighborhood; (3) standard deviation of pixel-wise gray-level distribution across nuclei, in turn capturing the underlying chromatin or chromosome patterns in nuclei; and (4) the average number of neighbor nuclei within a 50-pixel radius around individual nuclei, in turn reflecting the global spatial arrangement of nuclei. The top feature has previously shown to be prognostic or diagnostic for a number of other solid tumors. For instance, Lu et al. [11] found a significant association between the nuclei orientation disorder and overall survival in early stage estrogen receptor-positive (ER+) breast cancer. Nuclear polarity has also been implicated in the diagnosis and prognosis of urothelial [30] and papillary thyroid cancers [31]. The second feature, relating to spatial architecture of nuclei was found to over-express in high ODx patients compared to low and intermediate ODx patients. The patterns appear to suggest a more chaotic and disordered nuclear morphology in high ODx patients compared to the low and intermediate ODx patients. Interestingly, Whitney et al. [12] showed that these features were also discriminating of early-stage ER+ invasive breast cancer patients corresponding to high and low DCIS risk category patients. Additionally, a textural pattern within the individual nuclei was also found to be discriminating between the high and intermediate-low ODx risk categories, possibly reflecting differences in chromatin patterns. Nuclear texture has been previously found to be discriminating of malignant and benign breast lesions on histopathology [32]. Lu et al. [11] similarly found that differences in nuclear texture heterogeneity were associated with the overall survival for invasive breast cancer patients. Finally, the feature reflecting nuclei global spatial distribution implies that a high versus intermediate and low ODx risk category patients tended to have differences in clustering of nuclei in the proximity of necrotic regions on the slide. These findings appear to be aligned with the findings by Lagios et al. [33], which found that a higher concentration of necrosis was found to be associated with a higher risk of local recurrence for DCIS patients.

In experiment 3, we showed that the image features associated with ODx risk categories for DCIS were also found to independently distinguish between patients who progressed to invasive ductal carcinoma versus those who did not.

We envision two primary ways in which the image-based signature developed in this study might be used clinically. In developing countries or regions, where molecular-based assays like ODx test might not be easily affordable or even accessible for most of the DCIS patients, the imaging signature could potentially be employed as a surrogate of ODx test to prognosticate outcome, since the image-based assay is low-cost, non-tissue-destructive needing only digitized H&E slide images. Meanwhile, in developed countries, where molecular-based prognostic and predictive companion diagnostic tests exist, the image-based test could provide complementary morphologic cues to molecular and functional measurements of the tumor. The combination of computerized morphologic image attributes with an ODx risk score might help more accurately identify patients who could truly avoid adjuvant radiotherapy. This is in line with a recent study by Verma et al. [34] in early-stage ER+ invasive breast cancer, where the combination of an image-based morphologic predictor with the ODx assay was able to identify an additional 20% more patients who were truly low risk and could be spared adjuvant chemotherapy. Additionally, integrating ODx with our image-based assay could also provide additional improved characterization and stratification of those patients currently identified as intermediate risk by ODx.

Additionally, we also sought to evaluate the relative similarity in quantitative nuclear histomorphometric features between the intermediate ODx compared to low and high ODx risk categories. A higher AUC and a lower BC feature was obtained when grouping intermediate ODx together with low ODx as opposed to high ODx for any of the combinations of feature ranking methods and classifiers. Additionally, via the unsupervised clustering, the intermediate ODx risk category was found to be separable from high ODx risk category but not separable from low ODx risk category.

Taken in tandem, these results appear to suggest the histomorphometric features for intermediate ODx risk category patients were more similar compared to low ODx risk category patients as opposed to the high ODx risk patients. This is consistent to several recent studies in the context of invasive breast cancer [12, 35–37] that suggested that intermediate ODx risk category tumors appear to be more closely aligned with the low-risk tumors compared to the high ODx risk tumors. Kamal et al. [35] found that, based on the evaluation of traditional cancer prognosis criteria such as tumor size and tumor grade, invasive breast cancer in the high ODx risk category could be identified, but the discrimination between low and intermediate ODx risk categories could hardly be found. Also, a phase 3 clinical trial, TAILORx [36], concluded that for most patients with early-stage invasive breast cancer in intermediate ODx risk category, no benefit from receiving adjuvant chemotherapy could be observed in terms of overall survival as well as disease-free survival. In a study of the MammaPrint (MP) test (another widely used assay for invasive breast cancer), the study investigators found that among the patients in intermediate ODx risk category, most (65%) were identified as MP low-risk category [37], which is a category indicating low risk of recurrence for invasive breast cancer.

Our findings in light of the related previous studies [35–37] appear to suggest that intermediate ODx risk category patients appear to present very much like the low-risk patients and hence could possibly follow a similar management strategy.

This study did have some limitations. First, the sample size was too small to draw the definite conclusion that the intermediate ODx is comparable to low ODx risk category in terms of prognosis. Still, this study provides preliminary evidence that there is a quantifiable histomorphometric similarity between low and intermediate ODx risk category for DCIS. Although the impact of ODx test for DCIS on the clinical radiotherapy adoption had been confirmed by a study conducted by Manders et al. [8], the mismatch between low or high ODx risk category with the actual cancer aggressiveness still exists [7]. While we have independently evaluated the image signature associated with ODx risk categories to discriminate between patients who progressed to invasive ductal carcinoma as compared to those who did not in D2 (*n* = 30), clearly a larger multi-site cohort of DCIS patients is needed for definitive validation. Thirdly, the patients and image data were originated from a single facility, failing to take account of the tissue slide variance arisen from the slide preparation process as well as the differing patient population characteristics.

## Conclusions

In summary, computer-extracted quantitative nuclear histomorphometric features are able to distinguish between high ODx vs. low + intermediate DCIS ODx risk categories. Additionally, our findings suggest that based on computationally extracted nuclear histomorphometric features, the intermediate and low ODx risk categories were more similar compared to the high ODx risk category. Future work will involve independent validation of these findings on multi-site, multi-institutional data and also evaluating the ability of the histomorphometric features to identify DCIS patients at risk of progression to invasive disease.

## Supplementary information

**Additional file 1:** **Section S1.** Continuous ODx DCIS risk scores for each individual patients involved in D1.

**Additional file 2:** **Section S2.** The details on the tumor tissue slide preparation and scanning procedure (under the heading “Digital Slide Acquisition Procedure”). Normalized intensity distribution of cancer nuclei in the H&E stained slide images for patients in D1 (Fig. 1); multivariate (estrogen receptor status, progesterone receptor status and age) Cox proportional hazards analysis on risk class derived from ODx risk categories for the different tasks in D1 (Table 1); Detailed description of the features corresponding to each of the five feature families (under the heading “Feature Description”); Illustration of the feature maps within a tissue image of a DCIS patient corresponding to the high (I), intermediate (II) and low (III) ODx risk category (Fig. 3).

**Additional file 3:** **Section S3.** H&E stained slide tissue images for each of the three ODx risk categories in D1.

**Additional file 4:** **Section S4.** name list for all the extracted nuclear histomorphometric features.

## Data Availability

The datasets used and/or analyzed during the current study are available from the corresponding author on reasonable request.

## Abbreviations

AUC: Area under the receiver operating characteristic curve
BC: Bhattacharyya
CCG: Cell Cluster Graph
CORE: Cell Orientation Entropy
DCIS: Ductal carcinoma in situ
ER+: Estrogen receptor positive
ER: Estrogen receptor
H&E: Hematoxylin and eosin
Inter: Intermediate
IRB: Institutional review board
LDA: Linear discriminant analysis
MP: MammaPrint
MRMR-mid: Maximum relevance, minimum redundancy, Mutual Information Difference
MRMR-miq: Maximum relevance, minimum redundancy, Mutual Information Quotient
ODx: Oncotype DX
PCA: Principal component analysis
PHI: Protected health information
PR: Progesterone receptor
QDA: Quadratic discriminant analysis
QH: Quantitative histomorphometry
RT: Radiotherapy
SVM: Support vector machine
WRST: Wilcoxon rank sum test
WSI: Whole slide image
