# Supplementary material for: Quantitative nuclear histomorphometric features are predictive of Oncotype DX risk categories in ductal carcinoma in situ: preliminary findings
Source: Breast Cancer Res. 2019 Oct 17;21:114. doi: 10.1186/s13058-019-1200-6 (PMC6798488; doi:10.1186/s13058-019-1200-6)
Supplement: Supplementary file 2 — Additional file 2: Section S2. The details on the tumor tissue slide preparation and scanning procedure (under the heading “Digital Slide Acquisition Procedure”). Normalized intensity distribution of cancer nuclei in the H&E stained slide images for patients in D1 (Fig. 1); multivariate (estrogen receptor status, progesterone receptor status and age) Cox proportional hazards analysis on risk class derived from ODx risk categories for the different tasks in D1 (Table 1); Detailed description of the features corresponding to each of the five feature families (under the heading “Feature Description”); Illustration of the feature maps within a tissue image of a DCIS patient corresponding to the high (I), intermediate (II) and low (III) ODx risk category (Fig. 3). [file 13058_2019_1200_MOESM2_ESM.docx]

**Digital Slide Acquisition Procedure:**

In D1, among the 62 tissue slides, 22 slides were scanned on a Leica SCN400 Whole slide scanner at 20x magnification in Indiana University and 40 slides were scanned using a Philips Ultra Fast scanner at 40x magnification in the Institute for Pathology at Case Western Reserve University. The images were subsequently downsized to 20x to maintain a consistent magnification across all the slide images. All tissue was processed for histopathologic analysis according to standard anatomic pathology protocols in compliance with all College of American Pathologist (CAP) standards^1^. The tissue was fixed in 10% neutral buffered formalin and processed overnight in standard tissue processors. Slides were cut at 5 microns and stained on a Ventana automated staining system.

In order to evaluate the staining variation across the different tissue slides, we plotted out the intensity distribution of cancer nuclei in the H&E stained slide image for each of the 62 patients in D1. As may be seen in Figure1, the staining intensity was relatively uniform across all the slides.


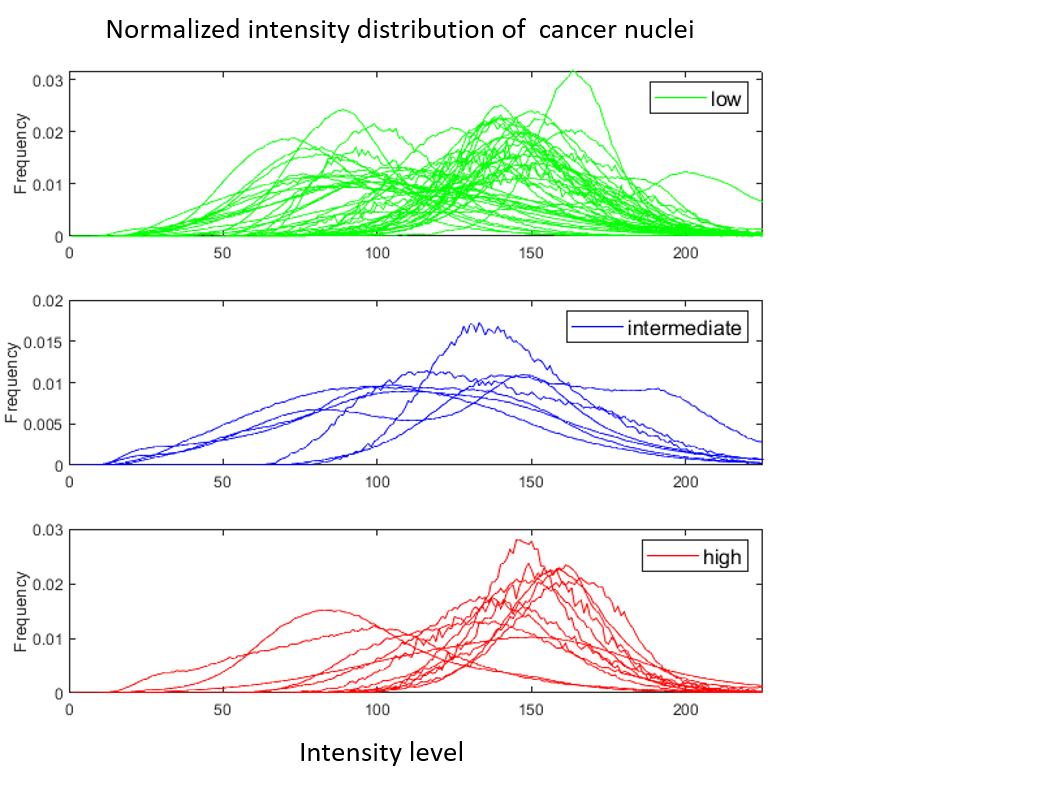


Figure 1: Normalized intensity distribution of cancer nuclei in the H&E stained slide images for patients in D1. The green curves (N=43) in the first plot represent patients corresponding to the low ODx risk category, blue curves (N=7) in the second plot represent patients corresponding to the intermediate ODx risk category and red curves (N=12) in the third plot represent patients corresponding to the high ODx risk category. The X axis corresponds to the gray level intensity of pixels, in turn corresponding to the cancer nuclei in the gray scale image converted from H&E stained slide images. The Y axis corresponds to the proportion of number of pixels with a certain intensity level compared to the summation of all the pixels.

Besides the univariate analysis, we have also performed a multivariate analysis based off three clinical variables with the ODx risk categories. Specifically, we built six different cox proportional hazards regression models using clinical variables (percentage of ER positively stained cells, percentage of PR positively stained cells and age) as predictors and the class derived from ODx risk categories for six different tasks (High vs. Low, High vs. Inter, Inter vs. Low, High vs. Inter + Low, High + Inter vs. Low, High vs. Inter vs. Low). The results including the p values, Hazard ratio along with the corresponding 95% confidential interval are shown in Table 1. No clinical variable was found to be significantly associated with the ODx risk categories in multivariate analysis.

|  | ER | PR | Age |
| --- | --- | --- | --- |
|  | P value / HR (95% CI) | P value / HR (95% CI) | P value / HR (95% CI) |
| High vs. Low | 0.61 / 0.95 (0.78-1.15) | 0.18 / 0.86 (0.69-1.07) | 0.82 / 0.99 (0.96-1.02) |
| High vs. Inter | 0.66 / 1.07 (0.76-1.52) | 0.33 / 0.74 (0.40-1.35) | 0.47 / 0.98 (0.93-1.03) |
| Inter vs. Low | 0.81 / 0.97 (0.77-1.22) | 0.55 / 0.93 (0.74-1.16) | 0.93 / 1.00 (0.97-1.03) |
| High vs. Inter + Low | 0.86 / 0.98 (0.81-1.18) | 0.12 / 0.84 (0.67-1.04) | 0.82 / 0.99 (0.97-1.02) |
| High + Inter vs. Low | 0.61 / 0.95 (0.80-1.13) | 0.24 / 0.88 (0.72-1.08) | 0.77 / 0.99 (0.96-1.02) |
| High vs. Inter vs. Low | 0.69/ 0.96 (0.80-1.15) | 0.07/ 0.81 (0.65-1.01) | 0.67 / 0.99 (0.96-1.02) |

Table1: multivariate (estrogen receptor status, progesterone receptor status and age) Cox proportional hazards analysis on risk class derived from ODx risk categories for the different tasks.

**Feature Description:**

**Global Graph:** Global graph features which reflect the nuclear architecture have previously shown to be prognostic for invasive breast cancer^2^ and lung cancer^3^. Global graphs were constructed by considering each nucleus as a node. The individual nodes were then connected via algorithms corresponding to the Voronoi Diagrams, Delaunay Triangles and Minimum Spanning Trees. Nuclear spatial arrangement and nuclei density measurements were subsequently derived from the connections between the edges and polygons of these constructed graphs. In addition, for each individual nucleus, architectural measurements such as average and standard deviation of distances to the nearest three, five and seven neighbor nuclei, and number of neighbor nuclei in the 10, 20, 30, 40 and 50 pixel radius around each node were extracted.

**Nuclear Shape:** Previous work has shown that nuclear shape features are associated with invasive breast cancer outcome^4^. Nuclear shape features were extracted from the nuclear boundaries to capture individual nuclear appearance. The 25 shape measurements consisted of nuclear area, perimeter, smoothness, fractal dimension, invariant moments, and Fourier descriptors of nuclear boundary points. The mean, median, standard deviation, and minimum/maximum ratio of these 25 measurements were calculated to give a total of 100 features.

**CORE:** The CORE feature family comprised quantitative measurements of the degree of disorder in nuclear orientation. These disorder measurements are derived from an 18 × 18 nuclear orientation co-occurrence matrix, with each row or column corresponding to a discretized nuclear orientation angle from 0 to 180 degrees with an interval of 10.

**CCG:** In the CCG feature family, the nuclei were clustered into different neighborhoods based on their spatial proximity to derive the measurements relating to local nuclear spatial arrangement, packing, and clustering. From the subgraph, the features including sub-graph radius, connectivity, and eccentricity were extracted. CCG features have been identified to be associated with the risk of recurrence in prostate cancer^5^.

**Haralick Texture:** A total of 26 Haralick texture features which correspond to heterogeneity patterns relating to the arrangement of the chromatin within the individual nuclei were also extracted.


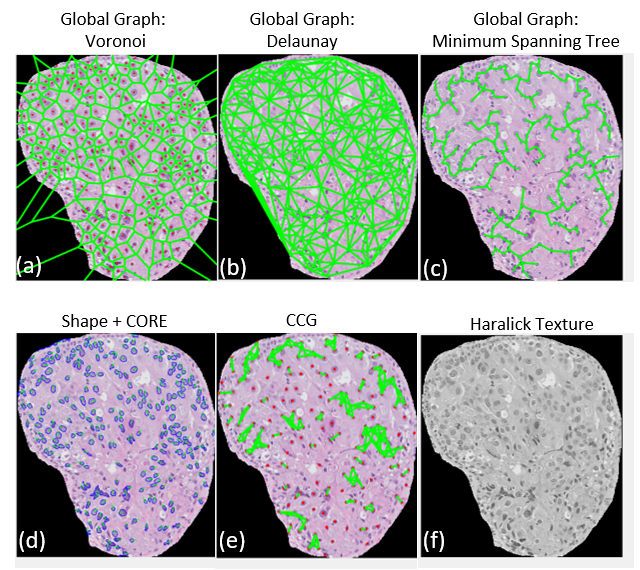


**(I)**


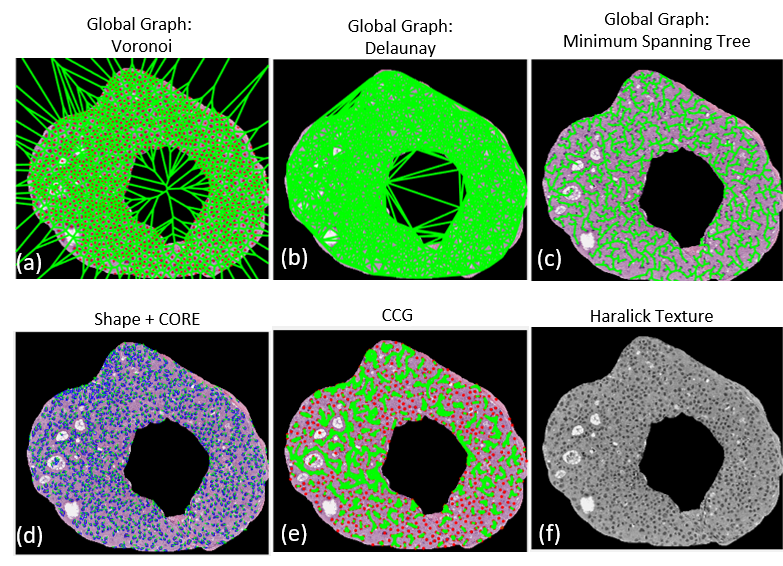


**(II)**


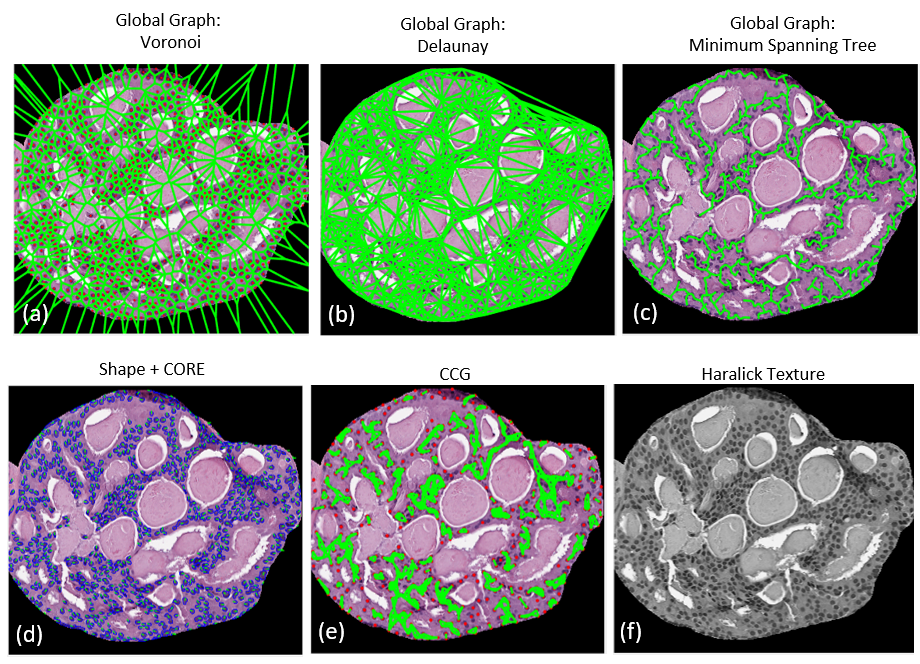


**(III)**

Figure 2: Illustration of the feature maps corresponding to global graph (Voronoi, Delaunay, and Minimum Spanning Tree), Shape, CORE, CCG and Haralick Texture features, capturing respectively spatial arrangement, shape, orientation, local arrangement and heterogeneity of nuclei within a tissue image of a DCIS patient corresponding to the high (I), intermediate (II) and low (III) ODx risk category respectively.

**Reference:**

1. National Society for Histotechnology. Guidelines_For_Hematoxylin_and_Eosin_Staining.pdf [Internet]. [cited 2019 May 25]. Available from: http://nsh.org/sites/default/files/Guidelines_For_Hematoxylin_and_Eosin_Staining.pdf

2. Whitney J, Corredor G, Janowczyk A, Ganesan S, Doyle S, Tomaszewski J, Feldman M, Gilmore H, Madabhushi A. Quantitative nuclear histomorphometry predicts oncotype DX risk categories for early stage ER+ breast cancer. BMC Cancer. 2018 May 30;18(1):610. doi:10.1186/s12885-018-4448-9 PMID: 29848291 PMCID: PMC5977541

3. Wang X, Janowczyk A, Zhou Y, Thawani R, Fu P, Schalper K, Velcheti V, Madabhushi A. Prediction of recurrence in early stage non-small cell lung cancer using computer extracted nuclear features from digital H&E images. Scientific Reports. 2017 Oct 19;7(1):13543. doi:10.1038/s41598-017-13773-7

4. Lu C, Romo-Bucheli D, Wang X, Janowczyk A, Ganesan S, Gilmore H, Rimm D, Madabhushi A. Nuclear shape and orientation features from H&E images predict survival in early-stage estrogen receptor-positive breast cancers. Lab Invest. 2018 Jun 29; doi:10.1038/s41374-018-0095-7 PMID: 29959421

5. Cell Orientation Entropy (COrE): Predicting Biochemical Recurrence from Prostate Cancer Tissue Microarrays | SpringerLink [Internet]. [cited 2018 Feb 6]. Available from: https://link.springer.com/chapter/10.1007%2F978-3-642-40760-4_50
