# Supplementary material for: Quantitative nuclear histomorphometric features are predictive of Oncotype DX risk categories in ductal carcinoma in situ: preliminary findings
Source: Breast Cancer Res. 2019 Oct 17;21:114. doi: 10.1186/s13058-019-1200-6 (PMC6798488; doi:10.1186/s13058-019-1200-6)

High ODx risk category (> 54)

ODx DCIS score: 70

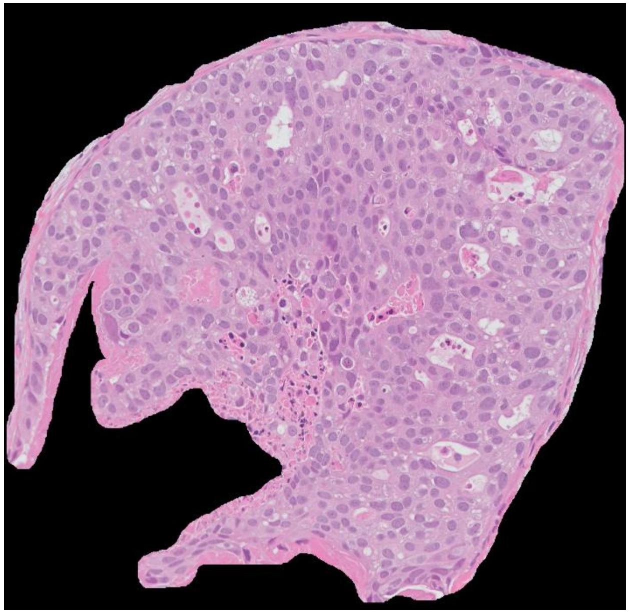

ODx DCIS score: 73

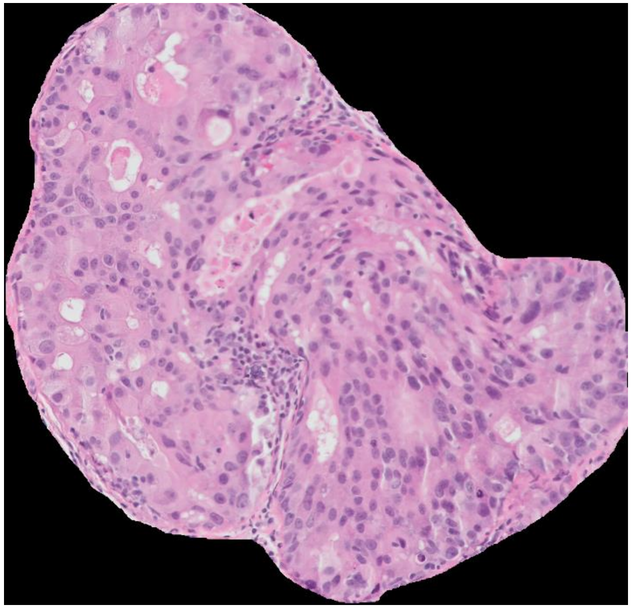

ODx DCIS score: 77

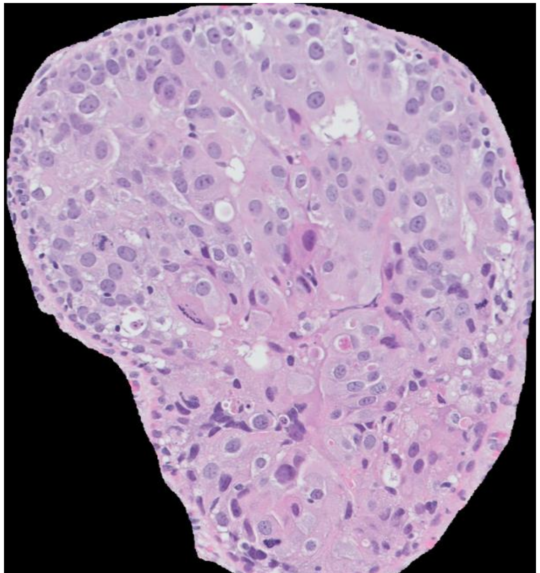

ODx DCIS score: 57

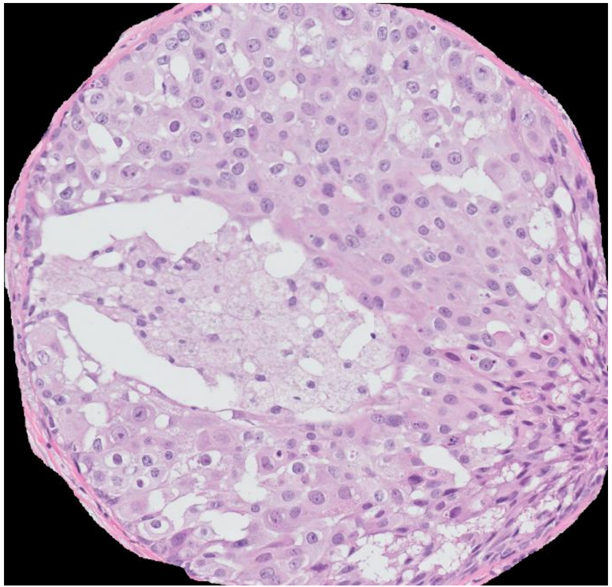

Intermediate ODx risk category (39 ~ 54)

ODx DCIS score: 50

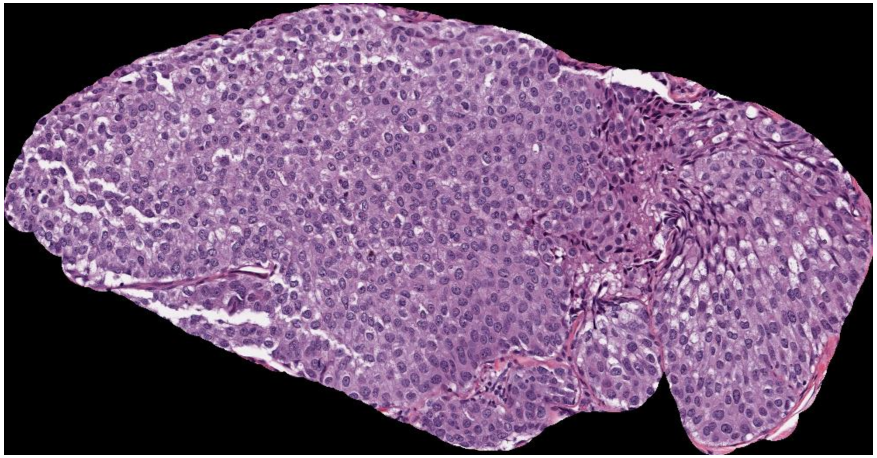

ODx DCIS score: 44

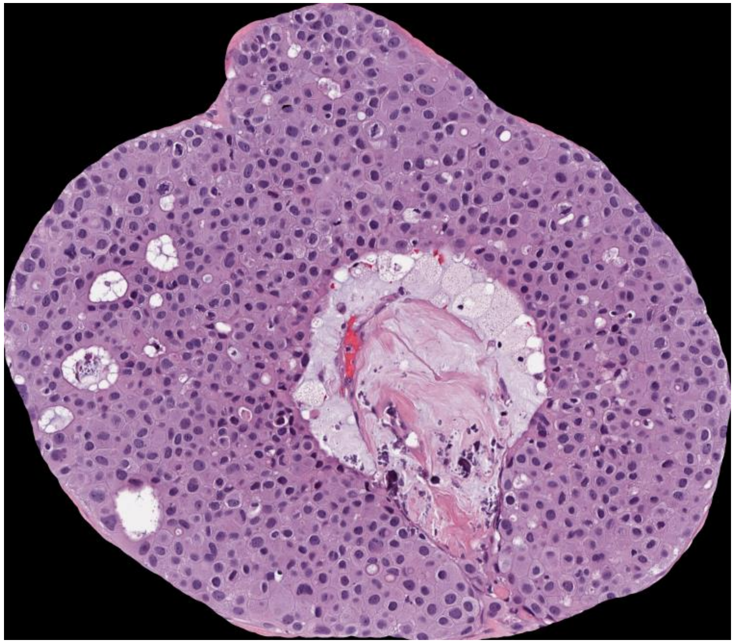

ODx DCIS score: 46

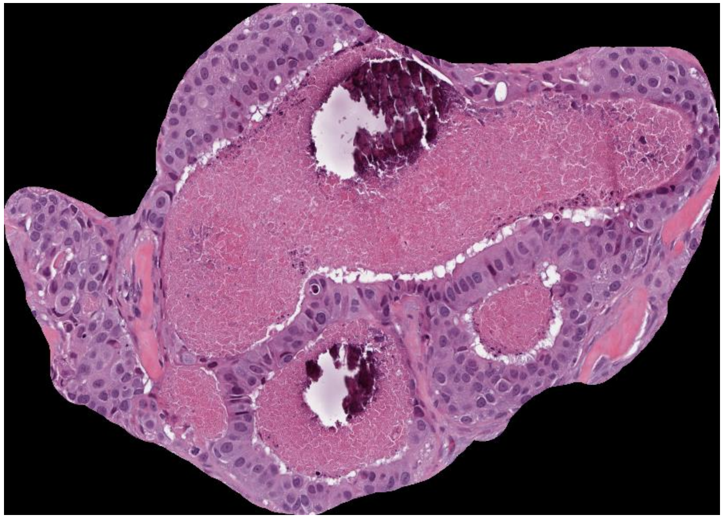

ODx DCIS score: 53

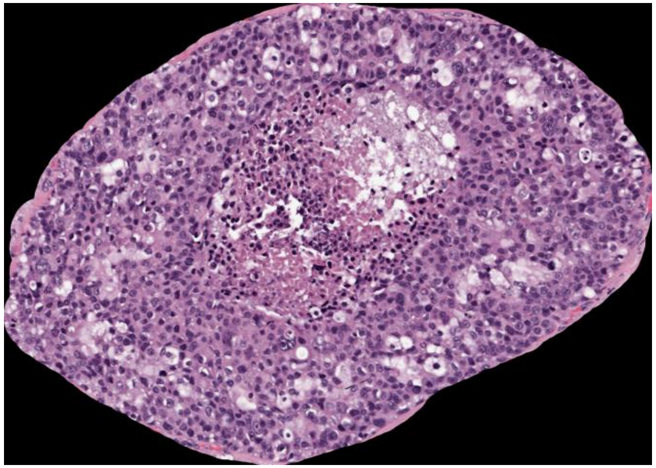

Low ODx risk category (<39)

ODx DCIS score: 0

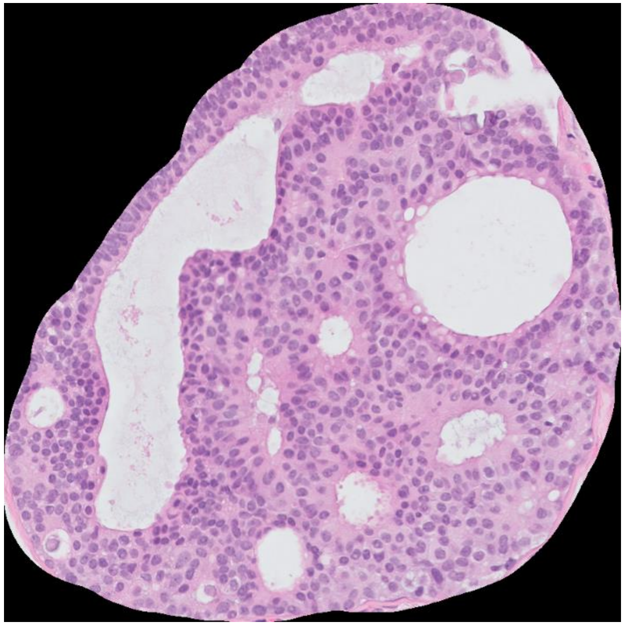

ODx DCIS score: 34

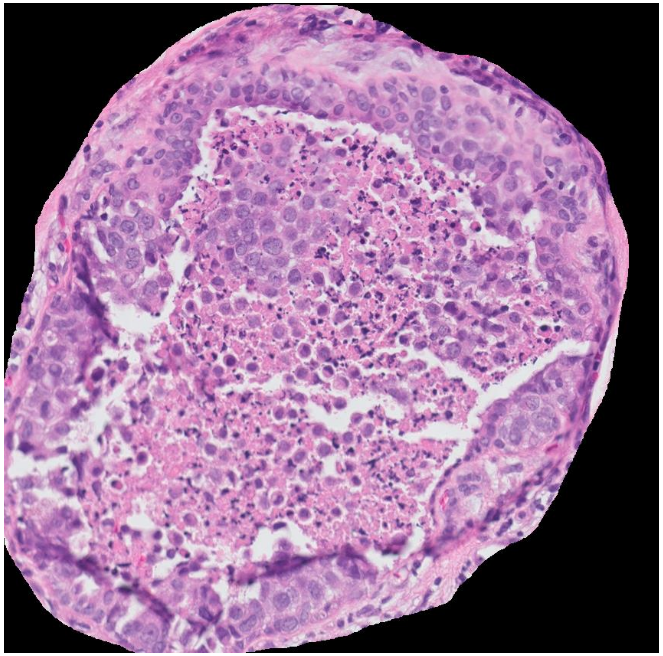

ODx DCIS score: 0

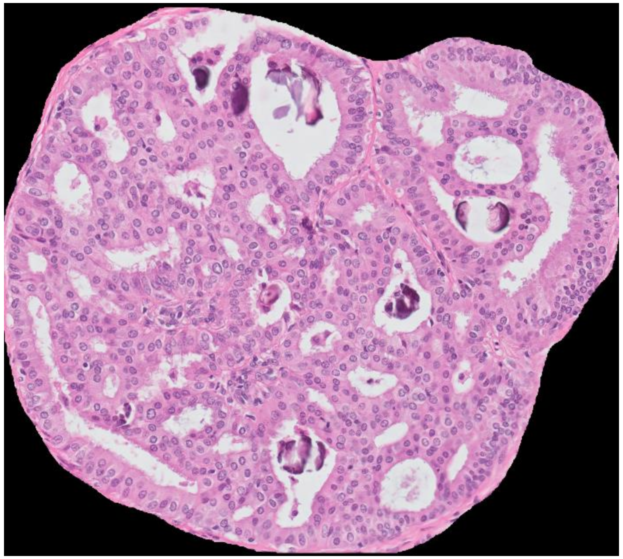

ODx DCIS score: 0

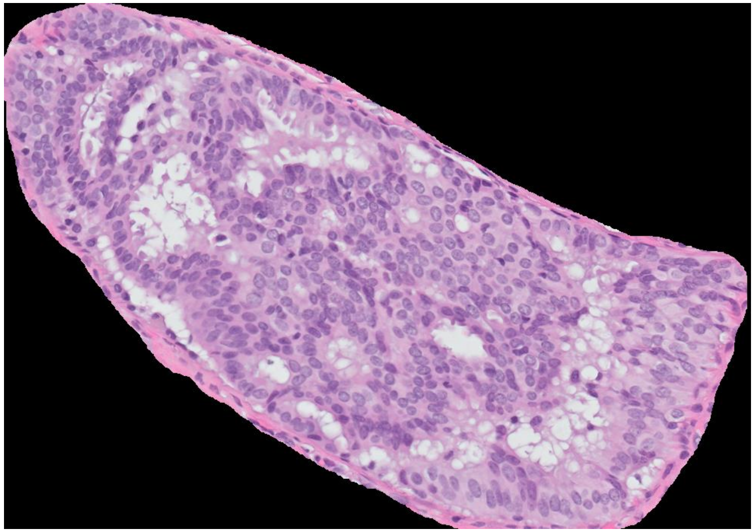

Supplement: Supplementary file 3 — Additional file 3: Section S3. H&E stained slide tissue images for each of the three ODx risk categories in D1. [file 13058_2019_1200_MOESM3_ESM.pdf]
